# Editorial

**Published:** 1902-09-15

**Authors:** 


### EDITORIAL.

THE annual meeting of the three national dental organizations were held this year at Niagara Falls, beginning with the Faculty Association and Examiners, on July 24th to 26th, and the National Dental Association, July 28th to 31st.. The attendance was large, as is usually the case when the meetings are held at this place. The weather was comfortable and much business was transacted. In the Faculty Association the time was largely taken up with the discussion of technical details in the management of the schools. No business of special interest to the profession was up for consideration. The committee on curriculum submitted a schedule of studies for the four year course which is to become operative next fall. The committee on foreign relations made a report as to the status of its work in the matter of the prosecution of schools and persons who are illegally engaged in trafficking in dental degrees. The chairman reported that one of these trials had just been completed in Chicago and a favorable decision was confidently anticipated. Since then this decision has been handed down and is as anticipated thoroughly satisfactory to the profession. The judge severely censuring the manager of the college concerned in this case. The association unanimously voted another assessment on the colleges to continue the work of prosecution until all such schools shall have been closed. It is costing the dental colleges a nice sum to do this work, but there seemed to be no disposition to let the work stop for lack of funds. The National Dental Association also added a contribution of five hundred dollars to continue the work.

There were an unusually large number of papers offered to the National Dental Association, but owing to the present cumbersome machinery through which they are forced to be brought out, not more than two-thirds were read, and some of those read received little or no discussion. But fifteen of the thirty-two clinics announced were given. The first section to report had six papers, and these consumed the first two days of the time, leaving a day and one half for the other four sections and the election of officers. The present plans of carrying on the program are manifestly unfair to the essayists and unsatisfactory to those who attend, and unless some change is made in the near future, this organization is doomed to lose the respect of the profession. A new constitution was submitted last year which it was hoped would be adopted at this meeting, and which, if adopted, would do much to make the general meetings of this body of interest and value to the profession, but it was laid on the table for consideration next year. The meeting is to be held at Asheville, N. C., next year. It is to be hoped that the executive committee and section officers will get together and agree upon a program which can be presented in the time at the disposal of the meeting, and that sufficient variety in the program may be given, so that all who attend may receive some satisfactory remuneration for the time and expense involved.

To the ranks of the profession the exhibits of appliances were of infinitely more value than the proceedings of the association. All the more prominent manufacturers of dental supplies were represented in a most satisfactory manner; each seemed to be vieing with the others to present his most attractive productions. There were new chairs, cabinets, electric engines, nitrous oxid appliances, porcelain outfits, gold preparations, cements, amalgams, new and old remedies, a new tin alloy for filling, crown and bridge-work appliances, electric mallets, matrixes, sterilizers, etc. The best of everything was to be seen, and their merits were fully elucidated by intelligent and courteous attendants.

The incidental entertainment of those in attendance was not provided by the profession of Niagara Falls, as it was last year by the dentists of Milwaukee and Wisconsin. There was no occasion, however, for this, as the place has sufficient in itself to occupy very profitably all the spare time at one's disposal. The various sights about the place are always attractive, even to those who have seen them many times. On Sunday afternoon the Buffalo Dental College gave all who cared to make the trip, a free ride to the mouth of the Niagara River by way of the Gorge Electric Railway. The visit to old Fort Niagara and a short sketch of its history by Dr. Barrett, was very interesting as well as delightful. The view of the "Rapids" on the return trip is one of the most interesting features of Niagara, while possessing little of the grandeur of the Falls, it has its charms which can only be appreciated by observations at close range, such as can be had from the car as it travels along close to the turbulent waters.

The opportunities for personal contact at these meetings are not to be despised, and they have a value not always appreciated. Men occupying important positions in the profession as State officers, teachers and delegates of local organizations, as well as distinguished scientists and technical workers are here assembled. They are most readily approached, and always willing and ready to give of that which has inspired them to become leaders of thought and action. So that after all we should not be disappointed if the literary program does not come off as advertised, or the clinics fail to come up to our ideals. Let us keep up our loyalty to the national organizations and let every one plan to attend as often as possible, and no one should feel content unless he has attended at least one such gathering.

